# Supplementary material for: Microbiological quality assessment of five common foods sold at different points of sale in Burkina-Faso
Source: PLoS One. 2022 Apr 14;17(4):e0258435. doi: 10.1371/journal.pone.0258435 (PMC9009693; doi:10.1371/journal.pone.0258435)
Supplement: S1 Table — (DOCX) [file pone.0258435.s001.docx]

| Locality | Geographic coordinates | Food type | Total Coliforms | Total aerobic mesophilic flora | Thermotolerants Coliforms | *E. Coli* | Coagulase-positive staphylococci | *Salmonella* | yeast and mould | Anaerobic Sulfito Reductive (ASR) | *Clostridium perfringens* | *Bacillus cereus* |
| --- | --- | --- | --- | --- | --- | --- | --- | --- | --- | --- | --- | --- |
| CIN | ***N11°07.184’***  ***W00°00.180’*** | Beans | ˂1E+01 | 7.0E+01 | ˂1E+01 | ˂1E+01 | ˂1E+02 | Absent | ˂1E+01 | - | - | - |
| CIN | ***N11°07.157’***  ***W00°00.133’*** | Beans | ˂1E+01 | 3.0E+01 | ˂1E+01 | ˂1E+01 | ˂1E+02 | Absent | ˂1E+01 | - | - | - |
| NIA | ***N10°16.894’***  ***W004°54.992’*** | Beans | ˂1E+01 | 1.3E+03 | ˂1E+01 | ˂1E+01 | ˂1E+02 | Absent | ˂1E+01 | - | - | - |
| NIA | ***N10°16.632’***  ***W004°55.216’*** | Beans | ˂1E+01 | ˂1E+01 | ˂1E+01 | ˂1E+01 | ˂1E+02 | Absent | ˂1E+01 | - | - | - |
| DAK | ***N11°00.397’***  ***W001°06.996’*** | Beans | ˂1E+01 | ˂1E+01 | ˂1E+01 | ˂1E+01 | ˂1E+02 | Absent | ˂1E+01 | - | - | - |
| DAK | ***N11°00.388’***  ***W001°06.988’*** | Beans | ˂1E+01 | 7.8E+03 | ˂1E+01 | ˂1E+01 | ˂1E+02 | Absent | ˂1E+01 | - | - | - |
| BOB | ***N11°10.763’***  ***W004°27.775’*** | Beans | ˂1E+01 | 5.0E+01 | ˂1E+01 | ˂1E+01 | ˂1E+02 | Absent | ˂1E+01 | - | - | - |
| BOB | ***N11°12.874’***  ***W004°17.172’*** | Beans | ˂1E+01 | 5.0E+01 | ˂1E+01 | ˂1E+01 | ˂1E+02 | Absent | ˂1E+01 | - | - | - |
| OUA | ***N12°20.019’***  ***W001°31.947’*** | Beans | ˂1E+01 | 9.0E+01 | ˂1E+01 | ˂1E+01 | ˂1E+02 | Absent | ˂1E+01 | - | - | - |
| OUA | ***N12°20.886’***  ***W001°31.827’*** | Beans | 1.0E+02 | 1.2E+04 | 4.0E+01 | 2.0E+01 | ˂1E+02 | Absent | ˂1E+01 | - | - | - |
| OUA | ***N12°20.826’***  ***W001°30.179’*** | Beans | ˂1E+01 | 5.6E+03 | ˂1E+01 | ˂1E+01 | ˂1E+02 | Absent | ˂1E+01 | - | - | - |
| OUA | ***N12°23.756’***  ***W001°27.508’*** | Beans | ˂1E+01 | 1.5E+04 | ˂1E+01 | ˂1E+01 | 2.9E+02 | Absent | 4.0E+01 | - | - | - |
| CIN | ***N11°07.152’***  ***W00°00.029’*** | Bread | 4.0E+01 | 2.5E+02 | ˂1E+01 | ˂1E+01 | ˂1E+02 | Absent | 1.0E+01 | - | - | - |
| CIN | ***N11°07.177’***  ***W00°00.141’*** | Bread | ˂1E+01 | ˂1E+01 | ˂1E+01 | ˂1E+01 | ˂1E+02 | Absent | ˂1E+01 | - | - | - |
| CIN | ***N11°07.018’***  ***W00°00.434’*** | Bread | ˂1E+01 | 1.0E+03 | ˂1E+01 | ˂1E+01 | ˂1E+02 | Absent | 2.0E+01 | - | - | - |
| NIA | ***N10°16.735’***  ***W004°55.092’*** | Bread | ˂1E+01 | 2.7E+02 | ˂1E+01 | ˂1E+01 | ˂1E+02 | Absent | 3.0E+01 | - | - | - |
| NIA | ***N10°16.164’***  ***W004°55.513’*** | Bread | ˂1E+01 | 6.5E+03 | ˂1E+01 | ˂1E+01 | ˂1E+02 | Absent | 3.7E+02 | - | - | - |
| NIA | ***N10°16.162’***  ***W004°55.579’*** | Bread | ˂1E+01 | 8.0E+01 | ˂1E+01 | ˂1E+01 | ˂1E+02 | Absent | ˂1E+01 | - | - | - |
| DAK | ***N11°00.611’***  ***W001°07.032’*** | Bread | ˂1E+01 | 2.0E+01 | ˂1E+01 | ˂1E+01 | ˂1E+02 | Absent | ˂1E+01 | - | - | - |
| DAK | ***N11°00.485’***  ***W001°07.019’*** | Bread | ˂1E+01 | 8.0E+01 | ˂1E+01 | ˂1E+01 | ˂1E+02 | Absent | ˂1E+01 | - | - | - |
| BOB | ***N11°11.204’***  ***W004°19.384’*** | Bread | ˂1E+01 | 7.0E+03 | ˂1E+01 | ˂1E+01 | ˂1E+02 | Absent | 5.1E+02 | - | - | - |
| BOB | ***N11°10.717’***  ***W004°17.823’*** | Bread | ˂1E+01 | 2.7E+02 | ˂1E+01 | ˂1E+01 | ˂1E+02 | Absent | 1.0E+01 | - | - | - |
| BOB | ***N11°10.324’***  ***W004°17.917’*** | Bread | ˂1E+01 | 7.9E+02 | ˂1E+01 | ˂1E+01 | ˂1E+02 | Absent | 2.0E+01 | - | - | - |
| OUA | ***N12°19.991’***  ***W001°31.962’*** | Bread | 1.8E+02 | 1.3E+03 | 4.0E+10 | ˂1E+01 | ˂1E+02 | Absent | 2.0E+02 | - | - | - |
| OUA | ***N12°21.247’***  ***W001°32.013’*** | Bread | ˂1E+01 | 6.5E+02 | ˂1E+01 | ˂1E+01 | ˂1E+02 | Absent | 5.3E+02 | - | - | - |
| OUA | ***N12°20.990’***  ***W001°31.878’*** | Bread | ˂1E+01 | 2.0E+01 | ˂1E+01 | ˂1E+01 | ˂1E+02 | Absent | ˂1E+01 | - | - | - |
| OUA | ***N12°20.837’***  ***W001°30.174’*** | Bread | ˂1E+01 | 3.8E+02 | ˂1E+01 | ˂1E+01 | ˂1E+02 | Absent | 1.0E+01 | - | - | - |
| CIN | ***N11°07.185’***  ***W00°00.180’*** | Pasta | ˂1E+01 | ˂1E+01 | ˂1E+01 | ˂1E+01 | ˂1E+02 | Absent | ˂1E+01 | - | - | - |
| CIN | ***N11°07.156’***  ***W00°00.133’*** | Pasta | ˂1E+01 | 8.0E+01 | ˂1E+01 | ˂1E+01 | ˂1E+02 | Absent | ˂1E+01 | - | - | - |
| NIA | ***N10°16.138’***  ***W004°55.549’*** | Pasta | ˂1E+01 | 1.1E+02 | ˂1E+01 | ˂1E+01 | ˂1E+02 | Absent | ˂1E+01 | - | - | - |
| NIA | ***N10°16.904’***  ***W004°54.995’*** | Pasta | ˂1E+01 | 3.4E+02 | ˂1E+01 | ˂1E+01 | ˂1E+02 | Absent | ˂1E+01 | - | - | - |
| DAK | ***N11°00.249’***  ***W001°07.066’*** | Pasta | 4.0E+01 | 1.4E+03 | 1.0E+10 | ˂1E+01 | ˂1E+02 | Absent | 4.2E+02 | - | - | - |
| DAK | ***N11°00.588’***  ***W001°06.970’*** | Pasta | ˂1E+01 | 1.3E+02 | ˂1E+01 | ˂1E+01 | ˂1E+02 | Absent | ˂1E+01 | - | - | - |
| BOB | ***N11°11.016’***  ***W004°21.996’*** | Pasta | ˂1E+01 | 1.6E+02 | ˂1E+01 | ˂1E+01 | ˂1E+02 | Absent | ˂1E+01 | - | - | - |
| BOB | ***N11°12.908’***  ***W004°17.173’*** | Pasta | ˂1E+01 | ˂1E+01 | ˂1E+01 | ˂1E+01 | ˂1E+02 | Absent | ˂1E+01 | - | - | - |
| BOB | ***N11°10.713’***  ***W004°17.824’*** | Pasta | ˂1E+01 | 2.0E+01 | ˂1E+01 | ˂1E+01 | ˂1E+02 | Absent | ˂1E+01 | - | - | - |
| OUA | ***N12°23.756’***  ***W001°27.508’*** | Pasta | ˂1E+01 | ˂1E+01 | ˂1E+01 | ˂1E+01 | ˂1E+02 | Absent | ˂1E+01 | - | - | - |
| OUA | ***N12°19.996’***  ***W001°31.994’*** | Pasta | ˂1E+01 | 1.0E+01 | ˂1E+01 | ˂1E+01 | ˂1E+02 | Absent | ˂1E+01 | - | - | - |
| OUA | ***N12°20.923’***  ***W001°31.876’*** | Pasta | 5.0E+01 | 4.9E+04 | 3.0E+01 | 3.0E+01 | ˂1E+02 | Absent | ˂1E+01 | - | - | - |
| CIN | ***N11°07.021’***  ***W00°00.438’*** | Rice+Sauce | ˂1E+01 | 6.0E+01 | ˂1E+01 | ˂1E+01 | ˂1E+02 | Absent | ˂1E+01 | - | - | - |
| CIN | ***N11°07.049’***  ***W00°00.413’*** | Rice+Sauce | ˂1E+01 | 2.9 E+03 | ˂1E+01 | ˂1E+01 | ˂1E+02 | Absent | ˂1E+01 | - | - | - |
| CIN | ***N11°07.146’***  ***W00°00.134’*** | Rice+Sauce | ˂1E+01 | 5.0E+01 | ˂1E+01 | ˂1E+01 | ˂1E+02 | Absent | ˂1E+01 | - | - | - |
| NIA | ***N10°16.089’***  ***W004°55.606’*** | Rice+Sauce | ˂1E+01 | 2.7E+02 | ˂1E+01 | ˂1E+01 | ˂1E+02 | Absent | ˂1E+01 | - | - | - |
| NIA | ***N10°16.098’***  ***W004°55.586’*** | Rice+Sauce | 1E+01 | 2.5E+03 | ˂1E+01 | ˂1E+01 | ˂1E+02 | Absent | ˂1E+01 | - | - | - |
| NIA | ***N10°16.910’***  ***W004°55.018’*** | Rice+Sauce | 1.1E+02 | 2.9E+04 | ˂1E+01 | ˂1E+01 | ˂1E+02 | Absent | 5.1E+03 | - | - | - |
| DAK | ***N11°00.374’***  ***W001°07.009’*** | Rice+Sauce | ˂1E+01 | 2.3E+02 | ˂1E+01 | ˂1E+01 | ˂1E+02 | Absent | ˂1E+01 | - | - | - |
| DAK | ***N11°00.249’***  ***W001°07.066’*** | Rice+Sauce | ˂1E+01 | 2.0E+01 | ˂1E+01 | ˂1E+01 | ˂1E+02 | Absent | ˂1E+01 | - | - | - |
| DAK | ***N11°00.465’***  ***W001°06.998’*** | Rice+Sauce | ˂1E+01 | 1.1E+02 | ˂1E+01 | ˂1E+01 | ˂1E+02 | Absent | ˂1E+01 | - | - | - |
| BOB | ***N11°11.016’***  ***W004°21.916’*** | Rice+Sauce | ˂1E+01 | 4.0E+01 | ˂1E+01 | ˂1E+01 | ˂1E+02 | Absent | ˂1E+01 | - | - | - |
| BOB | ***N11°11.553’***  ***W004°17.586’*** | Rice+Sauce | ˂1E+01 | 5.0E+01 | ˂1E+01 | ˂1E+01 | ˂1E+02 | Absent | ˂1E+01 | - | - | - |
| BOB | ***N11°12.906’***  ***W004°17.174’*** | Rice+Sauce | ˂1E+01 | 3.0E+04 | ˂1E+01 | ˂1E+01 | ˂1E+02 | Absent | 1.0E+01 | - | - | - |
| BOB | ***N11°11.148’***  ***W004°18.901’*** | Rice+Sauce | ˂1E+01 | 6.7E+03 | ˂1E+01 | ˂1E+01 | ˂1E+02 | Absent | ˂1E+01 | - | - | - |
| OUA | ***N12°19.061’***  ***W001°31.756’*** | Rice+Sauce | ˂1E+01 | 5.8E+03 | ˂1E+01 | ˂1E+01 | ˂1E+02 | Absent | ˂1E+01 | - | - | - |
| OUA | ***N12°20.608’***  ***W001°31.528’*** | Rice+Sauce | ˂1E+01 | 5.6E+03 | ˂1E+01 | ˂1E+01 | ˂1E+02 | Absent | ˂1E+01 | - | - | - |
| OUA | ***N12°21.184’***  ***W001°31.997’*** | Rice+Sauce | ˂1E+01 | 3.0E+01 | ˂1E+01 | ˂1E+01 | ˂1E+02 | Absent | ˂1E+01 | - | - | - |
| OUA | ***N12°25.131’***  ***W001°33.125’*** | Rice+Sauce | 2.0E+04 | 2.4E+05 | 1.7E+04 | ˂1E+01 | ˂1E+02 | Absent | 1.6E+04 | - | - | - |
| OUA | ***N12°22.557’***  ***W001°33.126’*** | Rice+Sauce | 7.9 E+02 | 5.5E+03 | 1.5E+02 | ˂1E+01 | ˂1E+02 | Absent | 1.0E+01 | - | - | - |
| OUA | ***N12°21.099’***  ***W001°28.802’*** | Rice+Sauce | 5.0 E+01 | 2.6E+03 | 3.0E+10 | ˂1E+01 | ˂1E+02 | Absent | 2.1E+02 | - | - | - |
| NIA | ***N10°16.837’***  ***W004°54.885’*** | Milk | ˂1E+01 | 1.8E+02 | ˂1E+01 | ˂1E+01 | ˂1E+02 | Absent | ˂1E+01 | ˂1E+01 | not detected | not detected |
| NIA | ***N10°16.908’***  ***W004°54.819’*** | Milk | ˂1E+01 | 3.2E+02 | ˂1E+01 | ˂1E+01 | ˂1E+02 | Absent | ˂1E+01 | ˂1E+01 | not detected | not detected |
| CIN | ***N11°07.029’***  ***W00°00.395’*** | Milk | ˂1E+01 | 2.3E+02 | ˂1E+01 | ˂1E+01 | ˂1E+02 | Absent | ˂1E+01 | ˂1E+01 | not detected | Detected |
| CIN | ***N11°07.138’***  ***W00°00.063’*** | Milk | ˂1E+01 | 1.3E+02 | ˂1E+01 | ˂1E+01 | ˂1E+02 | Absent | ˂1E+01 | ˂1E+01 | not detected | not detected |
| CIN | ***N11°07.152’***  ***W00°00.105’*** | Milk | ˂1E+01 | ˂1E+01 | ˂1E+01 | ˂1E+01 | ˂1E+02 | Absent | ˂1E+01 | ˂1E+01 | not detected | not detected |
| CIN | ***N11°07.027’***  ***W00°00.492’*** | Milk | ˂1E+01 | 2.8E+02 | ˂1E+01 | ˂1E+01 | ˂1E+02 | Absent | ˂1E+01 | ˂1E+01 | not detected | Detected |
| CIN | ***N11°07.122’***  ***W00°00.112’*** | Milk | ˂1E+01 | 4.5E+01 | ˂1E+01 | ˂1E+01 | ˂1E+02 | Absent | ˂1E+01 | ˂1E+01 | not detected | not detected |
| CIN | ***N11°07.121’***  ***W00°00.113’*** | Milk | ˂1E+01 | 2.0E+01 | ˂1E+01 | ˂1E+01 | ˂1E+02 | Absent | ˂1E+01 | ˂1E+01 | not detected | not detected |
| DAK | ***N11°00.249’***  ***W001°07.020’*** | Milk | ˂1E+01 | 2.0E+01 | ˂1E+01 | ˂1E+01 | ˂1E+02 | Absent | ˂1E+01 | ˂1E+01 | not detected | not detected |
| DAK | ***N11°00.663’***  ***W001°07.053’*** | Milk | ˂1E+01 | 2.5E+02 | ˂1E+01 | ˂1E+01 | ˂1E+02 | Absent | ˂1E+01 | ˂1E+01 | not detected | not detected |
| DAK | ***N11°00.374’***  ***W001°07.009’*** | Milk | ˂1E+01 | 2.0E+01 | ˂1E+01 | ˂1E+01 | ˂1E+02 | Absent | ˂1E+01 | ˂1E+01 | not detected | not detected |
| DAK | ***N11°00.374’***  ***W001°07.009’*** | Milk | ˂1E+01 | 2.5E+01 | ˂1E+01 | ˂1E+01 | ˂1E+02 | Absent | ˂1E+01 | ˂1E+01 | not detected | not detected |
| DAK | ***N11°00.658’***  ***W001°07.053’*** | Milk | ˂1E+01 | 3.0E+01 | ˂1E+01 | ˂1E+01 | ˂1E+02 | Absent | ˂1E+01 | ˂1E+01 | not detected | not detected |
| DAK | ***N11°00.658’***  ***W001°07.058’*** | Milk | ˂1E+01 | 1.0E+01 | ˂1E+01 | ˂1E+01 | ˂1E+02 | Absent | ˂1E+01 | ˂1E+01 | not detected | not detected |
| OUA | ***N12°22.533’***  ***W01°33.058’*** | Milk | ˂1E+01 | 4.0E+02 | ˂1E+01 | ˂1E+01 | ˂1E+02 | Absent | ˂1E+01 | ˂1E+01 | not detected | Detected |
| OUA | ***N12°22.651’***  ***W01°32.926’*** | Milk | ˂1E+01 | 5.3E+02 | ˂1E+01 | ˂1E+01 | ˂1E+02 | Absent | ˂1E+01 | ˂1E+01 | not detected | not detected |
| BOB | ***N11°10.809’***  ***W004°20.733’*** | Milk | ˂1E+01 | 1.5E+01 | ˂1E+01 | ˂1E+01 | ˂1E+02 | Absent | 1.0E+01 | ˂1E+01 | not detected | not detected |
| BOB | ***N11°11.157’***  ***W004°19.346’*** | Milk | ˂1E+01 | 6.2E+02 | ˂1E+01 | ˂1E+01 | ˂1E+02 | Absent | ˂1E+01 | ˂1E+01 | not detected | not detected |
| BOB | ***N11°11.087’***  ***W004°19.345’*** | Milk | ˂1E+01 | 2.0E+02 | ˂1E+01 | ˂1E+01 | ˂1E+02 | Absent | ˂1E+01 | ˂1E+01 | not detected | not detected |
| BOB | ***N11°10.598’***  ***W004°17.036’*** | Milk | ˂1E+01 | 4.0E+01 | ˂1E+01 | ˂1E+01 | ˂1E+02 | Absent | ˂1E+01 | ˂1E+01 | not detected | not detected |
| BOB | ***N11°10.634’***  ***W004°17.313’*** | Milk | ˂1E+01 | 3.8E+02 | ˂1E+01 | ˂1E+01 | ˂1E+02 | Absent | ˂1E+01 | ˂1E+01 | not detected | not detected |
| BOB | ***N11°12.030’***  ***W004°17.391’*** | Milk | ˂1E+01 | 1.1E+02 | ˂1E+01 | ˂1E+01 | ˂1E+02 | Absent | ˂1E+01 | ˂1E+01 | not detected | not detected |
| BOB | ***N11°12.001’***  ***W004°17.446’*** | Milk | ˂1E+01 | 2.3E+02 | ˂1E+01 | ˂1E+01 | ˂1E+02 | Absent | ˂1E+01 | ˂1E+01 | not detected | not detected |
| BOB | ***N11°09.683’***  ***W004°17.635’*** | Milk | ˂1E+01 | 5.5E+02 | ˂1E+01 | ˂1E+01 | ˂1E+02 | Absent | 1.0E+01 | ˂1E+01 | not detected | not detected |
| BOB | ***N11°09.821’***  ***W004°17.640’*** | Milk | ˂1E+01 | 4.3E+02 | ˂1E+01 | ˂1E+01 | ˂1E+02 | Absent | 1.5E+01 | ˂1E+01 | not detected | not detected |
| OUA | ***N12°19.988’***  ***W01°31.946’*** | Milk | ˂1E+01 | 3.0E+02 | ˂1E+01 | ˂1E+01 | ˂1E+02 | Absent | ˂1E+01 | ˂1E+01 | not detected | not detected |
| OUA | ***N12°20.057’***  ***W01°32.037’*** | Milk | ˂1E+01 | 3.3E+02 | ˂1E+01 | ˂1E+01 | ˂1E+02 | Absent | ˂1E+01 | ˂1E+01 | not detected | not detected |
| OUA | ***N12°19.081’***  ***W01°31.935’*** | Milk | ˂1E+01 | 1.1E+02 | ˂1E+01 | ˂1E+01 | ˂1E+02 | Absent | ˂1E+01 | ˂1E+01 | not detected | not detected |
| OUA | ***N12°21.103’***  ***W01°31.386’*** | Milk | ˂1E+01 | 2.2E+02 | ˂1E+01 | ˂1E+01 | ˂1E+02 | Absent | ˂1E+01 | ˂1E+01 | not detected | not detected |
| OUA | ***N12°20.520’***  ***W01°31.542’*** | Milk | ˂1E+01 | 4.1E+02 | ˂1E+01 | ˂1E+01 | ˂1E+02 | Absent | ˂1E+01 | ˂1E+01 | not detected | not detected |
| OUA | ***N12°20.715’***  ***W01°30.289’*** | Milk | ˂1E+01 | 2.8E+02 | ˂1E+01 | ˂1E+01 | ˂1E+02 | Absent | 6.0E+01 | ˂1E+01 | not detected | not detected |
| OUA | ***N12°23.826’***  ***W01°27.311’*** | Milk | ˂1E+01 | 3.6E+02 | ˂1E+01 | ˂1E+01 | ˂1E+02 | Absent | ˂1E+01 | ˂1E+01 | not detected | Detected |
| OUA | ***N12°23.873’***  ***W01°27.388’*** | Milk | ˂1E+01 | 1.8E+02 | ˂1E+01 | ˂1E+01 | ˂1E+02 | Absent | ˂1E+01 | ˂1E+01 | not detected | not detected |
| OUA | ***N12°21.072’***  ***W01°28.783’*** | Milk | ˂1E+01 | 2.0E+02 | ˂1E+01 | ˂1E+01 | ˂1E+02 | Absent | ˂1E+01 | ˂1E+01 | not detected | not detected |
| OUA | ***N12°21.096’***  ***W01°28.761’*** | Milk | ˂1E+01 | 1.7E+02 | ˂1E+01 | ˂1E+01 | ˂1E+02 | Absent | ˂1E+01 | ˂1E+01 | not detected | not detected |
| OUA | ***N12°20.995’***  ***W01°31.878’*** | Milk | ˂1E+01 | 1.7E+02 | ˂1E+01 | ˂1E+01 | ˂1E+02 | Absent | 1.0E+01 | ˂1E+01 | not detected | not detected |
| OUA | ***N12°25.162’***  ***W01°33.096’*** | Milk | ˂1E+01 | 3.2E+02 | ˂1E+01 | ˂1E+01 | ˂1E+02 | Absent | ˂1E+01 | ˂1E+01 | not detected | not detected |
| OUA | ***N12°21.617’***  ***W01°31.996’*** | Milk | ˂1E+01 | 2.2E+02 | ˂1E+01 | ˂1E+01 | ˂1E+02 | Absent | ˂1E+01 | ˂1E+01 | not detected | not detected |
| NIA | ***N10°16.938’***  ***W004°54.821*** | Milk | ˂1E+01 | 2.2E+02 | ˂1E+01 | ˂1E+01 | ˂1E+02 | Absent | 1.0E+01 | ˂1E+01 | not detected | not detected |
| NIA | ***N10°17.013’***  ***W004°54.843*** | Milk | ˂1E+01 | 1.4 E+02 | ˂1E+01 | ˂1E+01 | ˂1E+02 | Absent | ˂1E+01 | ˂1E+01 | not detected | not detected |
| NIA | ***N10°16.784’***  ***W004°55.038*** | Milk | ˂1E+01 | 2.2E+02 | ˂1E+01 | ˂1E+01 | ˂1E+02 | Absent | 1.0E+01 | ˂1E+01 | not detected | not detected |
| NIA | ***N10°16.304’***  ***W004°55.402*** | Milk | ˂1E+01 | 1.9E+02 | ˂1E+01 | ˂1E+01 | ˂1E+02 | Absent | ˂1E+01 | ˂1E+01 | not detected | not detected |
| NIA | ***N10°16.150’***  ***W004°55.529*** | Milk | ˂1E+01 | 1.6 E+02 | ˂1E+01 | ˂1E+01 | ˂1E+02 | Absent | 2.0E+01 | ˂1E+01 | not detected | not detected |

NIA : Niangoloko ; CIN : Cinkansé ; DAK : Dakola ; BOB : Bobo and OUA : Ouagadougou.
