# Supplementary material for: Microbiological quality assessment of five common foods sold at different points of sale in Burkina-Faso
Source: PLoS One. 2022 Apr 14;17(4):e0258435. doi: 10.1371/journal.pone.0258435 (PMC9009693; doi:10.1371/journal.pone.0258435)
Supplement: Attachment — Submitted filename: Reviewer_attachments.docx [file pone.0258435.s003.docx]

L.18 - please specify to which category these types of foods belong: raw or ready-to-eat

L.25: Please specify the reference of the guide values used to judge the compliance of the samples

L.24-26: Is there a scientific reason why the percentage of contaminated food should be expressed to the nearest hundredth?

L.25, L.30, L.31: Do you mean "satisfactory" as compared to "m" or "M"? Please specify.

Table 1: Switch "," to ".". - A subtotal of raw and ready-to-eat is an added value.

L.103: ISO 6887: XXXX. Please specify the year of the standard followed.

L.107, L.116...The standards mentioned have been cancelled or revised. Please indicate the bibliographic reference justifying your choices.

L.112, L.116: An incubation period of 24 to 48 hours is noted? Specify which duration was used for the study. The 2 durations are not comparable in terms of enumeration.

L.168: ISO7932:1993 is an enumeration method and not a research method. Please change the expression of the results in table 2.

Table 2: The criteria chosen are from Dunod, 2003. [19]. These are not international guideline values. Please specify other bibliographic references using the same reference for comparison of the compliance percentages obtained.

In the context of a study related to public health, what about the conformity of the samples?

You would verify the target "m" for E. coli and thermotolerant coliforms. These are the same

A.185: Have you verified the homogeneity within each type of food, since the points of sale are different. Is there a significant difference between the sampled locations?

Table 3: Missing details on the volume seeded for staphylococci and yeast/molds that impact the limit of quantification. Switch "<1" to "<10" or "<100" as appropriate. This also applies to bacillus cereus. For search methods, use "detected" or "not detected."

A.229: Specify the reference of the origin of the guide values.

What does "similar" mean? What are the results reported by references [4 and 21].

L. 234: "The highest... "Please indicate the results of the distribution (mean, max., min, median...) in the results section if you wish to discuss them.

L. 241: Differentiate between raw and ready-to-eat results. The average concentration of total flora is to be interpreted differently.

L. 242: Stay with your original idea. The total flora is an indication of good conservation, of hot or cold chain.

L. 250 to 252: there is a difference between the % of contamination by coliforms and E. coli. You do not specify the source that indicates that total coliforms are related to fecal contamination. It seems to me that E. coli is the indicator of fecal contamination.

A261 to 263: Reread reference [1]. Staphylococci are primarily indicators of hand hygiene and good hand washing practices.
