# Supplementary material for: Microbiological quality assessment of five common foods sold at different points of sale in Burkina-Faso
Source: PLoS One. 2022 Apr 14;17(4):e0258435. doi: 10.1371/journal.pone.0258435 (PMC9009693; doi:10.1371/journal.pone.0258435)
Supplement: Attachment — Submitted filename: PONE-D-21-30741_comments_M Badrul Amin.docx [file pone.0258435.s004.docx]

Microbiological quality assessment of five common foods sold at different
points of sale in Burkina-Faso

The study is important from food safety perspectives where microbiological quality assessment has been focused on five different foods in five regions of Burkina Faso. The study is interesting though the sample size is not big enough to draw a conclusion about the food safety issue in different areas of Burkina Faso. The abstract of the manuscript is incomplete considering the findings as there is a lack of information such sampling localities, foods sampling sites (restaurants or street food), bacterial count (colony forming unit, cfu) etc. Besides, how many samples were contaminated with hygiene and safety indicator organisms? In methods, EMB agar is not selective for only E. coli, other gram-negative bacteria might grow. How did author confirm E. coli colony through EMB agar culture? There is no cfu count limit of each parameters tested in methods sections. The results were described partially and no information about the count of hygiene and safety indicators in text. The abstract shows that 11.88% foods are satisfactory in terms of microbiological standards where the results show 24% food samples were contaminated with yeast and molds, which is contradictory. In my opinion, this manuscript is not well organized. We have observed many spelling mistakes and grammatical errors throughout the manuscripts. The following comments should be addressed throughout the manuscript:

- All percentage data should be provided with number. e.g. 43 (13%) or 13% (n=43).
- The word ‘satisfactory/appreciation was used in multiple places of the manuscript but there is no definition of it. Is it according to international food safety criteria or guidelines?
- The sample collection method was not described in the manuscript, only the sites of collection and sample types were mentioned.
- In line 20, 101 is not required to write.
- In line 23 & 24, ‘All samples were analyzed under ISO methods’ should be ‘Samples were tested according to ISO guidelines for all parameters.
- Sampling period was not mentioned in ‘Materials and methods’ section.
- In line 23, ‘*Bacillus cereus* were checked too’ should be ‘*Bacillus cereus* were also tested for contamination’.
- In line 30, recorded” should be placed in lieu of “record”
- In line 39-42, there is no reference for “According to……diarrhea to cancers” statement.
- Line 59: “Illnesses” should be placed in lieu of “illness”.
- Line 66-68 “In developing countries…..capital is required” is not clear to me. Rewording is required. Drove in the sentence should be driven.
- Lin 71, not proper style of citation. Suggestion: according to previous study or something like that.
- Line 78: Common” should be placed in lieu of “commons
- Line 90: are should be were.
- In line 92, ‘Figure 1 indicated the sampling localities.’ Should be ‘Figure 1. Sample collection sites in the map of Burkina Faso.’
- Line 97, parameters name should be mentioned. And “are” should be “were”.
- Line 100-101, homogenized in water and again homogenized in mixer bag. How many times you homogenized?
- In line 101, add time and rotation per minute for homogenization in bag mixer.
- In line 102, ‘with in’ should be ‘with’.
- Line 107, what is PCA?
- In line 108, replace ‘was’ with ‘were’.
- In line 108, 112, 116, 121 it was not mentioned why the plates with low number of colonies were selected.
- In line 112, ‘Only the Petri dish’ should be ‘culture plates’
- In line 116, ‘Only the Petri dish’ should be ‘culture plates’.
- Line 124, what is IMViC?
- Line 128, positive clones or colonies? What is BBLTM?
- In line 129, remove ‘agar’ after ‘(EMB)’ and ‘France’ should be in the bracket.
- In line 130, “was used”: should be written.
- Footnote is missing for table 2. what is m or M? what is UFC?
- Use ‘CFU/g’ instead of in ‘UFC/g’ throughout the manuscript according to ISO guidelines.
- What is the use of statistical analysis in Methods section? Application of significance test described in study analysis is missing in entire manuscript. Suggestion: please remove this form methods.
- In line 134, they mentioned sesame seeds but they are not using any sesame samples. please remove sesame.
- In line 142, ‘purified on’ should be ‘streaked onto’.
- In line 142, remove ‘then submitted to’ and add ‘were performed using API 20E (BioMérieux, France) test’. How many colonies were undergone for API 20E biochemical tests for each parameter. Please mention in detail in the methods section.
- In line 144, “key” and “included” should be used instead of “Key” and “including”
- In line 145, replace ‘H2S’ with ‘H_2_S’.
- In line no. 149 pepton should be replaced with peptone.
- In line 150, replace ‘seeded’ to ‘spread’.
- In line 157, remove ‘full stop’ after ‘(Liofilchem diagnostic, Italy)’.
- In line 175, it was not defined what appreciation criteria is.
- Line 188, please mention criteria of what? is it food safety criteria according to international guidelines or regulation? which food items were found to be contaminated?
- Line 194, it is difficult to say pathogenic e. coli without performing molecular test like PCR.
- In Table 3, data for all samples is not necessary to present in details. Data could be presented graphically. Title of Table 3 is not complete, it should be more elaborative.
- In line 205, “one sample” should be written instead of “sample one”
- Line 210, what do mean by “the milk samples represent the highest percentage i.e., 42.57%”.
- In line no. 211 of result section “record” should be “recorded”
- Line 217, how 11.88% food samples were satisfactory where 24.7% were contaminated with yeast and molds.
- Reference missing in the statement “Total….wind” from line 232-234 .
- Line 235, 10^5^ up to10^9^ should be 10^5^ to10^9^
- Line 236, [24] report that…. This is not appropriate style of citing. Suggestion: according to previous report or something like that.
- In line no 239 of discussion section “Foods samples” should be “food samples”.
- In line 239, it should be written Except instead of Excepted
- In line 247, it will be 6.93% and 9.90%. entire manuscripts had these errors.
- Line 250, Coliforms should be colifomrs
- Entire manuscripts show percentage of satisfactory or appreciation. Number of samples found to be contaminated should be mentioned in the parentheses along with %.
- Line 253, signifies should be signify.
- Line 254, According to [6] is not proper style of referencing.
- There was no reference against the statement “That should…..other studies”; line 241-242.
- The rationale behind the statement “That suggest the …..pathogens” (line 251-252) was not clear.
- Unit of the count was missing in line 262 after 2.6×10^2^
- In line 269 it should be written, they appear to be present in higher number….
- In line 277, it should be written “previous studies find out….”
- In line 284, it will be 9.30%.
- Line 284 and 287, either word or number should be written. Ex. Four or 4. No need to write both.
- Line 290, expertise..?
- Line 317-319: The statement “A void that was necessary…. areas and urban center” is not clear.
- Line 324-326: too ambitious conclusion which is difficult to evaluate from this study findings. Suggestions: removed.
- They have stated “One might believe that the advent of covid-19 that has profoundly destabilized developing countries people and fundamentally changed their habits and behaviors might have contributed to reduce the contamination of food through handling.” from line 300-302, but they don't have any data to compare with the situation before covid 19.

**------------The End-----------**
