# Supplementary material for: Microbiological quality assessment of five common foods sold at different points of sale in Burkina-Faso
Source: PLoS One. 2022 Apr 14;17(4):e0258435. doi: 10.1371/journal.pone.0258435 (PMC9009693; doi:10.1371/journal.pone.0258435)
Supplement: Attachment — Submitted filename: Response to Reviewers.docx [file pone.0258435.s005.docx]

**Answers**

The authors would like to deeply thanks all the PLOS ONE board member for the best platform they provide for microbiological sciences promotion. Without any favor, it will be and honor for us if this manuscript is accepted for publication in PLOS ONE.

Authors also deeply thank the two reviewers for their valuable and pertinent critics that aim to improve the quality of the manuscript. We are already satisfied with the work done and will tried to bring answers for all the remarks they pointed out. Authors hope these answers will get reviewers approval.

**Additional answers**

1. Samples were collected from market restaurants for cooked foods (beans, rice with sauce, pasta) and from shops for milk and bread samples.

We cannot provide lot numbers of these food as they are artisan produced or bulk collected foods. GPS coordinates have been added for each sample in Supporting Information files.

2. Grant information have been updated to match with ‘Funding Information’ and ‘Financial Disclosure’ sections
3. Supporting Information files have been including.
4. Abstract on the online submission form (via Edit Submission) and abstract in the manuscript have been update.
5. Figure 1 have been removed and figure 3 (previous figure 4) is referred in the text at line 229.
6. Figure 1 have been removed from the manuscript.

Thank you very much

**Reviewer 1**

All observations of reviewer 1 have been taken into account

Line 210, “the milk samples represent the highest percentage i.e., 42.57%” mean that portion of milk sample over all sample is 42.57%

Line 217, how 11.88% food samples were satisfactory where 24.7% were contaminated with yeast and molds: 11.88% is the overall appreciation and 24.7% is appreciation focused in only yeast and molds.

**Reviewer 2**

All observations of reviewer 2 have been taken into account.

L.24-26: There is no scientific reason why the percentage of contaminated food is expressed to the nearest hundredth.

L.107, L.116...The standards mentioned have been cancelled or revised. Our laboratory still used these standards because we did not buy new standard yet.

A.185: Have you verified the homogeneity within each type of food, since the points of sale are different: we did not verify the homogeneity within each type of food and I think it will not be possible to do so.
